# Clinical application of human *β-defensin* and *CD14* gene polymorphism in evaluating the status of chronic inflammation

**DOI:** 10.1186/1479-5876-10-S1-S9

**Published:** 2012-09-19

**Authors:** Wings TY Loo, Lan-jun Bai, Chang-bin Fan, Yuan Yue, Yi-ding Dou, Min Wang, Hao Liang, Mary NB Cheung, Louis WC Chow, Jin-le Li, Ye Tian, Liu Qing

**Affiliations:** 1 UNIMED Medical Institute, Hong Kong SAR; 2 School of Chinese Medicine, Li Ka Shing Faculty of Medicine, The University of Hong Kong, Pokfulam, Hong Kong SAR; 3 Department of Stomatology, Sichuan Academy of Medical Sciences & Sichuan Provincial People’s Hospital, No.32, Section 2, 1st Ring Road (West), Chengdu, Sichuan Province, PRC; 4 Stomatological Hospital of Guangzhou Medical College, Guangzhou, PRC; 5 State Key Laboratory for Oral Diseases and Department of Prosthodontics, West China Hospital of Stomatology, Sichuan University, Sichuan, PRC; 6 Jin Hua Dentistry, Chengdu, 610041, Sichuan, PRC; 7 Keenlink Dental Clinic, Hong Kong SAR

## Abstract

**Background:**

Periodontitis is a common disease that affects the periodontal tissue supporting the teeth. This disease is attributed to multiple risk factors, including diabetes, cigarette smoking, alcohol, pathogenic microorganisms, genetics and others. Human beta-defensin-1 (hBD-1) is a cationic antimicrobial peptide with cysteine-rich ß-sheets and broad-spectrum antimicrobial activity. CD14 is a protein involved in the detection of bacterial lipopolysaccharide (LPS) and has also been associated with periodontitis. This study investigates the single nucleotide polymorphic (SNP) region, -1654(V38I), of the human beta-defensin-1 (hBD-1) gene as well as the -159 region of the *CD14* gene in subjects with chronic periodontitis.

**Methods:**

Blood samples from periodontally healthy subjects and periodontitis patients were obtained. DNA was extracted from the blood and was used to perform restriction digest at the polymorphic G1654A site of *DEFB1* with the enzyme *HincII.* The polymorphic site 159TT of *CD14* was digested with the enzyme *AvaII.* Enzyme-linked immunosorbent assay (ELISA) was performed on soluble samples to determine the protein expressions.

**Results:**

The control and patient groups expressed 35% and 38% 1654 A/A genotype of *DEFB1*, respectively. The A allele frequency of the control group was 40%, while the patient blood group was 54%. The mean hBD-1 protein levels of the control and patient samples were 102.83 pg/mL and 252.09 pg/mL, respectively. The genotype distribution of *CD14* in healthy subjects was 16% for C/C, 26% for T/T and 58% for C/T. The genotype frequencies of *CD14* in periodontitis patients were 10% for C/C, 43% for T/T and 47% for C/T. The CD14 protein expression determined by ELISA showed a mean protein level of the control samples at 76.28ng/mL and the patient blood samples at 179.27ng/mL with a *p* value of 0.001.

Our study demonstrated that patients suffering from chronic periodontitis present more commonly with the 1654A/A genotype on the *DEFB1* gene and the 159T/T genotype on the *CD14* gene.

**Conclusions:**

This study purely investigated the association between periodontitis and one polymorphic site on both *DEFB1* and *CD14* gene, with the purpose of expanding knowledge for the future development in diagnostic markers or therapeutic interventions to combat this disease.

## Background

Periodontitis is a chronic infectious disease[1] involving the degradation and destruction of periodontal supporting tissue of the teeth[2]. This disease is highly prevalent and can affect up to 90% of the worldwide population[3]. The causes and risk factors include oral microorganisms such as *Porphyromonas gingivalis* (*P. gingivalis*) [4], genetic factors (Nares, 2003), acquired environmental factors like tobacco smoking [5], uncontrolled diabetes [6], stress [7], impaired host response [3,8], alcohol use, HIV and AIDS, malnutrition, and osteoporosis[10]. Of the mentioned factors, genetic polymorphisms have attracted much attention, and a number of studies have been conducted in different ethnic populations worldwide to gain additional knowledge.

In recent years, a number of genetic polymorphisms and their association with periodontal diseases have been studied[11], which include interleukin (IL) -1 [12], IL-1α [13], IL-1β [14] IL-10 (Sumer *et al*., 2007)[15], IL-17 [16], matrix metalloproteinases [17] and others. Human β-defensins (hBDs) are a group of low molecular weight (3 to 5 kDa) cationic antimicrobial peptides with cysteine-rich ß-sheets. Defensins possess broad-spectrum antimicrobial activity that kill microbes by inducing physical holes in the membrane (Li *et al*., 2007)[18]. They contribute to host innate immunity by disrupting the membrane integrity of a broad spectrum of microorganisms [19]. These peptides, encoded by the *DEFB* genes, are present in three main gene clusters located on chromosome 8p22-34 [20]. At least four types of defensins (hBD-1 to hBD-4) have been characterized in humans. hBD-1 was first isolated from the hemofiltrate of patients with end stage kidney disease undergoing dialysis, and renal epithelia were found as the major source [21]. Human ß defensins, namely hBD-1 and hBD-2, have been recently identified from plasma and various epithelial tissues [21,22]. Although α and ß defensins are encoded by different genes, these genes share a common evolutionary origin [23]. hBD-2 expression is induced by stimuli such as bacteria and proinflammatory cytokine TNF-α, thereby evoking the important functions of hBD-2 in acute inflammation [23].

hBD-1 (encoded by *DEFB1*) and hBD-3 (encoded by *DEFB103*) are constitutively expressed at low levels in the skin [24]. hBD-1 is not normally expressed in the circulation. hBD-2 (encoded by *DEFB4*) is not expressed in normal skin but is highly expressed in inflammatory tissues. hBD-2 is absent or at low levels in normal epithelia. Serum hBD-2 levels in healthy individuals are very low (less than 1 ng per ml) [25]; conversely, the levels are highly inducible and expressed locally or systemically on inflammatory stimulation [26]. The localizations of hBD-2 expression include nasal [27] and oral mucosa [28], gingival epithelia [29], human airway [30], nasolacrimal duct [31], and ocular surface epithelium [32], as well as intestinal epithelium [33] in response to infection and inflammation. The constituent protein, hBD-1, is not normally expressed in the circulation. However, hBD-2 is inducible and expressed locally or systemically on stimulation [26].

Genetic polymorphisms of hBD-1 and their associations with periodontitis have been investigated at SNP sites -692 and -44, respectively [34]. The conducted studies concluded that associations do not exist for hBD-1 polymorphisms at these SNP sites and periodontitis. In contrast, SNP site -1654 of *DEFB1* has been demonstrated to have a positive correlation with common diseases such as chronic obstructive pulmonary disease [35] and atopic dermatitis [36]. However, its association with periodontitis has not been studied.

*CD14* is a 55-kDa glycosyl phosphatidylinositol-anchored glycoprotein expressed on the surface of white cells [37]. As a LPS receptor it can bind to the LPS, leading to nuclear factor-κB activation and cytokine expression mediated by the TLR4/MD2 complex [38]. *CD14* exists in two forms: either anchored to a cell membrane by a glycosylphosphatidylinositol tail (mCD14), or in soluble form (sCD14). sCD14 is produced by enzymatically cleaved membrane *CD14*, mediated mainly by phospholipase C, and via secretion of *CD14 *[39]. Usually, *CD14* binds to LPS in the presence of lipopolysaccharide-binding protein. Subsequently, a series of reactions take place and eventually trigger intracellular signalling, causing the production of pro-inflammatory cytokines. Its concentration in serum has been found to increase in several clinical pathologies, such as periodontitis [40], rheumatoid arthritis [41], systemic lupus erythematosus [42] and tuberculosis [43]. A SNP is found at position -159 in the *CD14* promoter, where a C to T transition occurs [59]. Previous studies have shown an association of the *CD14*-159 TT genotype with chronic periodontitis [40], myocardial infarction [44] and pulmonary tuberculosis [45].

Our recent *in vitro* study in the reconstituted human gingival epithelium model shows that in addition to TLR2 and TLR4, *CD14* may also be involved in the regulation of hBD-2 expression by *P. gingivalis* LPS [46]. Lipopolysaccharide, interleukin-1β (IL-1β), and tumor necrosis factor-a (TNF-α) are known as highly specific *β*-defensin inducers [19].

Considering the association of *CD14* genetic polymorphisms with the severity of chronic periodontitis [40], the present study investigated for the first time the potential association of both hBD and *CD14* polymorphisms and their serum levels with chronic periodontitis in Chinese subjects.

## Methods

### Selection of subjects

108 systemically healthy, non-smoking Chinese adults (39 females and 69 males, aged 18 to 60) were randomly selected from the voluntary blood donors at Hong Kong Red Cross between September 2004 and March 2007. After the blood samples of these healthy volunteers were collected, they were scheduled for oral examinations at Keenlink Dental Clinic, Hong Kong, where they were determined to be clinically healthy or have gingivitis, without furcation involvement or generalized gingival recession. All subjects who were determined to be free of oral soft tissue abnormalities or severe dental caries were allocated to the control group.

44 Chinese subjects (18 females and 26 males, aged 18 to 74 years) with moderate to advanced chronic periodontitis were recruited from West China Hospital of Stomatology, Sichuan University. The diagnosis of chronic periodontitis was made following the criteria defined by the American Academy of Periodontology in 1999 [49]. The subject data are presented in Table 1. Both groups of the subjects did not have more than 4 teeth missing in the dental ridge. They did not have systemic diseases (such as diabetes, uncontrolled hypertension), and they did have a smoking history.

**Table 1:** Demographic and clinical data (Mean ± SD; % of Subjects or Sites)

Parameters	Control subjects (N = 108)	Periodontitis patients (N = 44)
Age (years)	42.9 ± 9.7	49.3 ± 13.6
Age range (years)	18 - 60	18 - 74
Male/female	64/36	59/41
PD (mm)	2.7 ± 1.2	6.1 ± 2.7*
Sites% with BOP	40.3 ± 9.5	78.2 ± 19.8*
Sites% with gingival recession	1 ± 1.2	38.9 ± 25.9*
Sites% with calculus	34.1 ± 13.6	63.0 ± 25.8
Clinical Attachment Loss (mm)	0.0	6.2 ± 2.9

Significant difference from the control, * *p* < 0.05

The sample size of this study was determined based on the reports of Machin and Lemeshow. In the report, the sample size was determined based on 0.05 level of significance for two arms to achieve 90% power [47,48].

The study protocols were approved by the Ethics Committee, Faculty of Medicine, The University of Hong Kong and Sichuan University, PRC. Informed consent was obtained from all subjects.

### Sampling

Peripheral blood samples were collected by direct venipuncture from the arm vein of each subject: 20 ml in lithium heparin tubes and 10ml in clot blood tubes (BD Vacutainer, NJ USA), respectively. The samples were certrifuged for 10 min at 1,500 rotations per minute (rpm), and serum and plasma was then collected for enzyme-linked immunosorbent assay (ELISA) analysis. The remaining cellular components were transferred to a 50 ml centrifuge tube with an addition of red blood cell lysis buffer up to 45 ml. The mixture in the tube was inverted several times and then centrifuged for 10 min at 1,500 rpm. The supernatants were discarded, and the remaining components were washed with 0.9% PBS used for DNA extraction.

### Extraction of DNA

Genomic DNA was extracted from each blood sample using the QIAamp DNA Blood Mini Kit (QIAGEN, MD, USA). The DNA concentration was estimated by measurement of OD_260_. The extracted DNA was labelled and stored at -80°C until further analysis.

### Polymerase chain reaction (PCR), restriction enzyme cleavage and gel electrophoresis

The 111 bp exon region consisting of the G1654A SNP region of *DEFB1* was amplified using the following primers: forward, 5’-CAAGCCATGAGTCTGAAGTGT-3’; and reverse, 5’-TCAACAGTGGAGGGCAATGT-3’ according to a previous study[35]. A PCR kit (Promega Corporation, U.S.A) was used according to the manufacturer’s instructions. The kit consisted of a PCR Master Mix (50 units/ml Taq DNA Polymerase) supplied in a proprietary reaction buffer (pH 8.5), 400 μM each of dATP, dGTP, dCTP, dTTP, and 3 mM of MgCl_2_, and nuclease free water. All procedures were carried out in a sterile and stable environment to prevent external contamination. PCR was undertaken in a thermal cycler (MJ, U.S.A.) with a mixture containing 20 units of nuclease-free water, 25 units of Master Mix, 0.5 units of each primer and 3 units of extracted DNA sample. The cycling conditions programmed were denaturation at 94°C for 5 min, followed by 30 cycles at 94°C for 30 sec, 30 sec at 56°C, extension at 72°C for 1 min, with a final extension at 72°C for 5 min. The products from the thermal cycling were stored at -80°C until restriction enzyme digestion [35].

The 111 bp of *DEFB1* fragment generated from the PCR procedure was digested using *HincII* restriction enzyme (Fermentas Life Sciences, USA). A 10 μl aliquot of the PCR product was mixed with 5 units of *HincII*, 10 μl of nuclease free water and 1 μl of restriction enzyme buffer. The entire mixture was incubated at 37°C for 2 hours. All digestion reagents were kept on ice before incubation.

The electrophoresis was performed with 5 μl of digestion product and 1μl of Ready-Load 1 Kb DNA Ladder (Invitrogen, Spain) which were loaded onto a 4% agarose gel (Invitrogen, Spain), and the gel was visualized with ethidium bromide [35].

The analysis of -159 polymorphism of *CD14* was performed following a previous protocol [50]. The *CD14* gene promoter was amplified by a PCR fragment of about 500 base pairs of the following primers: forward, 5’-GTGCCAACAGATGAGGTTCAC-3’; and reverse, 5’-GCCTCTGACAGTTTATGTAATC-3’. PCR was performed with 250 ng DNA in Master Mix (Promega, WI, USA), 12.5 µl of each pairs of primers (15 pmol). The PCR conditions were denaturation at 94°C for 5 min, then 30 cycles at 94°C for 30 sec, 57°C for 30 sec, extension at 72°C for 1 min, and followed by a final extension at 72°C for 5 min. The products were stored at -80°C until restriction enzyme digestion. The PCR products was then digested by *AvaII* (Fermentas Life Sciences, USA) at 37°C for 16 h and separated in a 2.5% agarose gel. The gel underwent electrophoresis at 100 volts, 100 milliAmperes for 30 min. Visualization was performed by means of a Dolphin-DOC ultraviolet illuminator (Wealtec, South Africa).

### Assay of hBD-2 and CD14 by ELISA

The supernatant of the blood samples were used for hBD-2 and CD14 assays (ELISA kits from Phoenix Pharmaceuticals Inc, USA and DIACLONE, Besançon, France, respectively), following the manufacturer’s instructions. 100 µl of serum samples were pipetted into a 96-well microplate for assay of hBD-2 and CD14, respectively. The microplates were incubated at 350 rpm for 2 hours and washed with washing buffer three times. The wells were then dried and 200 μL of substrate tetramethylbenizidine was added into each well for 20 min at room temperature. The plates were then read at 450 nm wavelength using Universal Microplate Reader (Sunrise, TECAN, Austria). The levels of hBD-2 and CD14 were determined by comparison with the standard curve generated from the standards supplied by the manufacture. Each sample was analysed in triplicates. The levels of hBD-2 and CD14 were presented as pg/ml and ng/ml, respectively.

### Statistical analysis

The detection frequency and genotype distribution of *DEFB1* and *CD14* polymorphisms in the patient and control groups was compared by the Chi-square test. The relevant odds ratios between the groups were analyzed. The difference in serum levels of hBD-2 and CD14 between the groups were evaluated with an independent t-test. A *p*-value of 0.05 or less was regarded as statistically significant. Statistical analysis was performed using SPSS 15.0 for Windows (SPSS Inc., Chicago, IL, U.S.A.).

## Results

Table 1 details the clinical results of the periodontitis patient and the healthy control groups. The periodontitis patient group showed significantly greater means than the healthy control group (*p*<0.05) for the following clinical results: pocket depth (PD): 6.1 ± 2.7mm vs. 2.7 ± 1.2mm; Clinical Attachment Loss (CAL): 6.2 ± 2.9 mm vs. 0; percentage of sites with bleeding on probing (BOP): 78.2 ± 19.8% vs. 40.3 ± 9.5% and gingival recession: 38.9 ± 25.9% vs. 1 ± 1.2%. There was no significant difference found in the age and gender ratio between the groups.

The blood count of the control subjects and periodontitis patients are presented in Table 2. The results were within the normal range, although the patient group showed a relatively higher count and percentage of lymphocytes and a relatively lower count and percentage of neutrophils (*p* < 0.05) compared to the healthy control group.

**Table 2:** Blood count (Mean ± SD) of the control subjects and periodontitis patients

Parameters	Control subjects (N = 108)	Periodontitis patients (N=44)	Normal range	Unit
White blood cell	5.03(±1.25)	3.96(±1.02)	4.00-11.00	10^9^/L
Red blood cell	5.29(±0.11)	4.25(±0.10)	3.8-6.0	10^12^/L
Hemoglobin	15.9(±0.47)	12.5(±0.43)	11.5-16.5	g/dl
Platelet	256(±25.08)	241(±23.31)	150-400	10^9^/L
Neutrophil	3.67(±1.02)	*1.68(±0.71)	2.0-7.5	10^9^/L
Lymphocyte	1.29(±0.58)	*1.42(±0.26)	1.30-3.5	10^9^/L
Monocyte	0.44(±0.17)	0.18(±0.11)	0.2-0.7	10^9^/L
Eosinophil	0.11(±0.55)	0.06(±0.02)	0.0-0.5	10^9^/L
Basophils	0.02(±0.02)	0.02(±0.01)	0.0-0.1	10^9^/L

Significant difference from the control, * *p* < 0.05

**Figure 1:**
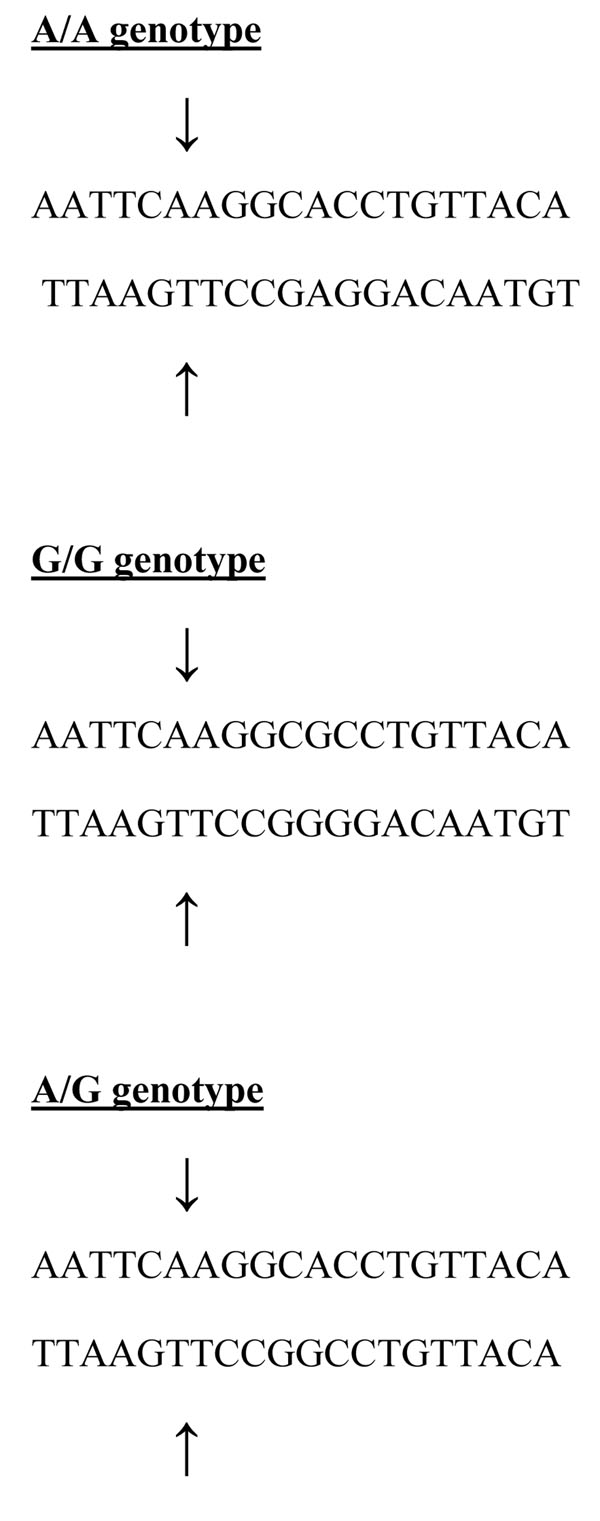
The arrows show the *HincII* restriction enzyme digestion at 111 bp position of *DEFB1* DNA polymorphism representing A/A, G/G and A/G genotypes.

**Figure 2:**
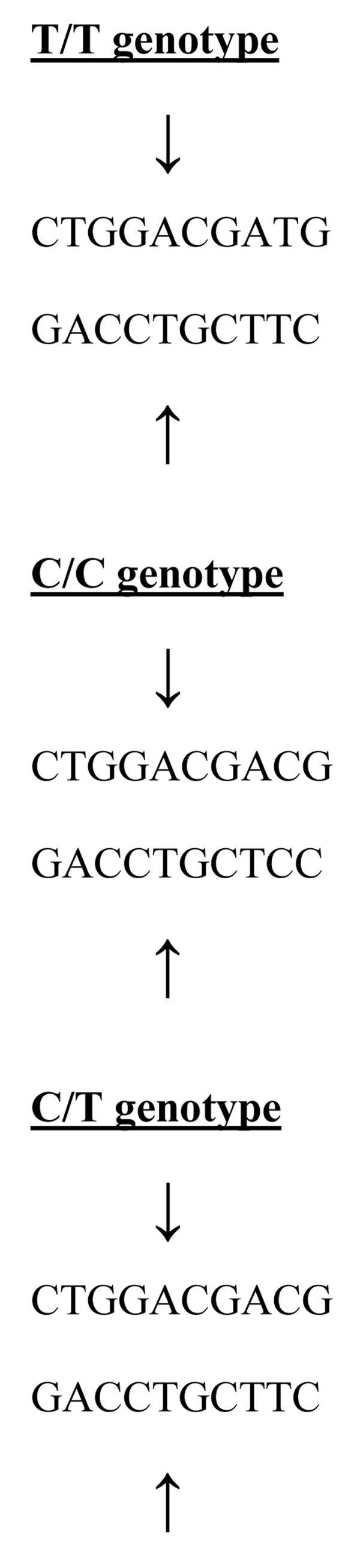
The arrows show the *AvaII* restriction enzyme digestion at -159 bp promoter region of *CD14* DNA polymorphism representing T/T, C/C and T/C genotypes.

**Figure 3:**
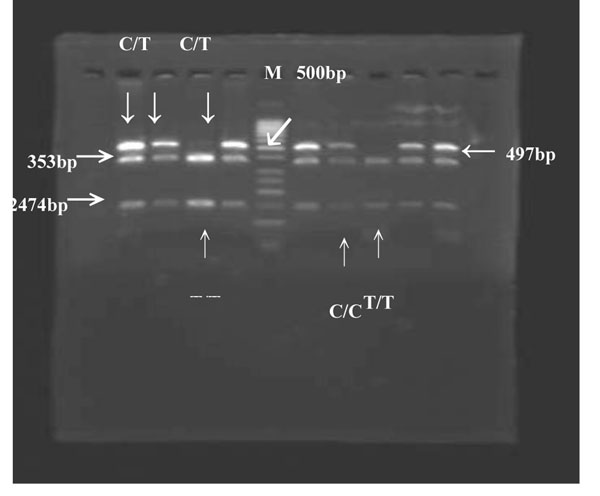
The DNA polymorphism of CD14 at position -159 digested with restriction enzyme *AvaII*. The homozygous C/C alleles were represented by a DNA band with a size of 497 bp (1 band, no cut); homozygous T/T alleles were represented by DNA bands with sizes 353 bp and 144 bp (2 bands), whereas heterozygotes displayed a combination of C/T alleles (3 bands: 497 bp, 353 bp and 144 bp). M represented the DNA marker.

The DNA sequence of *DEFB1* and *CD14* demonstrating where SNPs occur and restriction sites are shown in Figures 1, 2 and 3. For *DEFB1*, homozygous G/G alleles were represented by a DNA band with a size of 111 bp, and the homozygous A/A alleles were represented by DNA bands with sizes 93 and 18 bp. Heterozygotes displayed a combination of both alleles (111, 93, and 18 bp).

Homozygous C/C alleles of *CD14* were represented by a DNA band with a size of 497 bp, and the homozygous T/T alleles were represented by DNA bands with sizes of 353 and 114 bp. Heterozygotes C/T displayed a combination of the alleles (497, 353 and 144 bp). Overall, significant difference was found in the distribution of *DEFB1* and *CD14* genotypes between the groups (Table 3). For *DEFB1*, the detection frequency of G allele was significantly lower in the patient group (26%) than in the controls (59%) (*p* < 0.001). The subjects with the G allele are four-folded at lower risk for moderate to severe chronic periodontitis (*p* < 0.001, OR = 4.111 with 95% CI 2.378 – 7.107). The genotype of G/G was significantly lower in the patient group (20%) than the healthy controls (54%) (*p* < 0.001). Individuals with the G/G genotype are at approximately four times lower risk for moderate to severe periodontitis than people with A/A and G/A genotypes (*p* < 0.001, OR = 4.511 with 95% CI 1.988 – 10.288).

**Table 3:** Genotype and allele distribution of DEFB1 and CD14 in control subjects and periodontitis patients

Genotypes	CP Patients *n*=44 (%)	Healthy subjects *n*=108 (%)	CP versus Healthy subjects		Alleles	CP patient *n*=88 (%)	Healthy subjects *n*=216 (%)	CP versus Healthy subjects	
			
			OR (95% CI)	*p* values				OR (95% CI)	*p* values
***DEFB1***									
**A/A**	30 (69)	38 (35)	3.9474 (1.8697-8.3339)	<0.0002*	**A**	65 (74)	88 (41)	4.1107 (2.3775-7.1073)	<0.0001*
G/G	9 (20)	58 (54)			G	23 (26)	128 (59)		
G/A	5 (11)	12 (11)							
***CD14***									
**T/T**	19 (43)	28 (26)	2.1714 (1.0406-4.5311)	<0.0368*	**T**	59 (67)	119 (55)	1.6584 (0.9868-2.7869)	<0.05501
C/C	4 (10)	17 (16)			C	29 (33)	97 (45)		
C/T	21 (47)	63 (58)							

*DEFB1*: G/G+G/A versus A/A; *CD14*: C/C+C/T versus T/T; OR, odds ratio; CI, confidence interval

For *CD14*, the genotype of T/T was significantly higher in the patient group (43%) than the control group (26%) (*p* < 0.05). Individuals with the T/T genotype are at twice a greater risk to develop moderate to severe periodontitis than people with C/C and C/T genotypes (*p* < 0.05, OR = 2.171, 95% CI 1.041 – 4.531).

**Figure 4:**
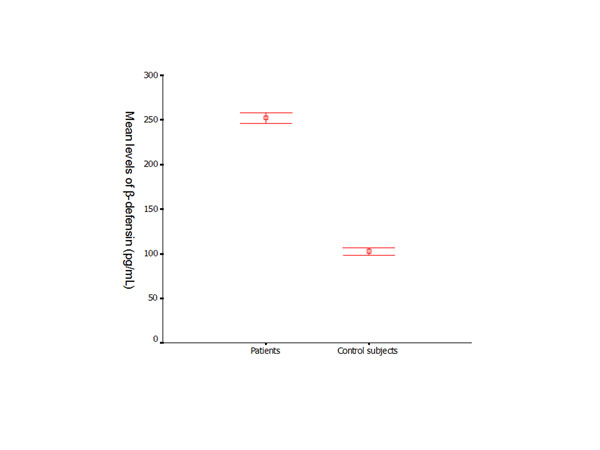
Serum levels of hBD-2 (Mean ± SD) in control subjects and periodontitis patients were measured by ELISA.

**Figure 5:**
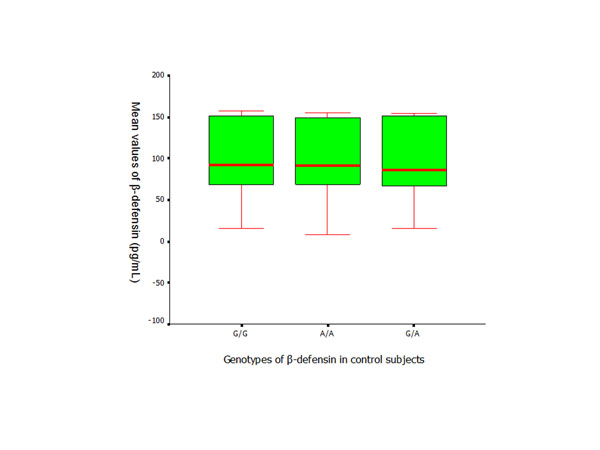
ELISA assay of hBD-2 (Mean ± SD) applied to measure the serum levels in control subjects with different *DEFB1* genotypes.

**Figure 6:**
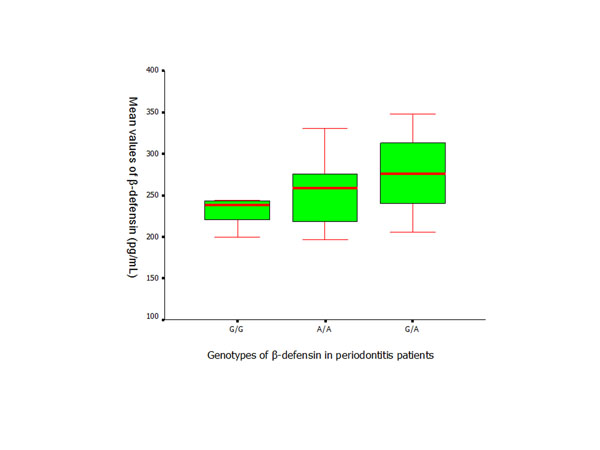
ELISA assay of hBD-2 (Mean ± SD) applied to measure the serum levels in periodontitis patients with different *DEFB1* genotypes.

The serum levels of hBD-2 in the patient group were significantly higher than the levels of healthy controls (*p* < 0.01) (Figure 4). Similar results were found between the subjects with the same genotypes from the control and patient groups. There was no significant difference found in the serum levels of hBD-2 within the control and patient groups who presented with different *DEFB1* genotypes (Figures 5 and 6).

**Figure 7:**
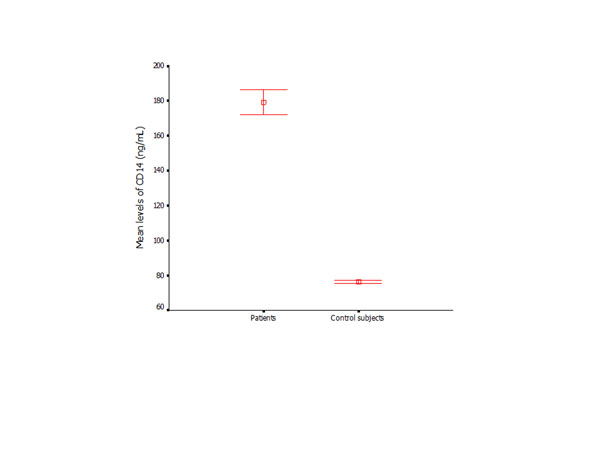
Serum levels of CD14 (Mean ± SD) in control subjects and periodontitis patients measured by ELISA.

**Figure 8:**
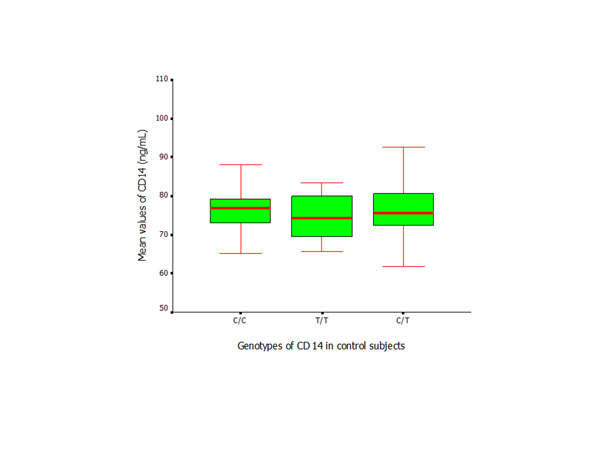
ELISA assay of CD14 (Mean ± SD) applied to measure the serum levels in control subjects with different *CD14* genotypes.

**Figure 9:**
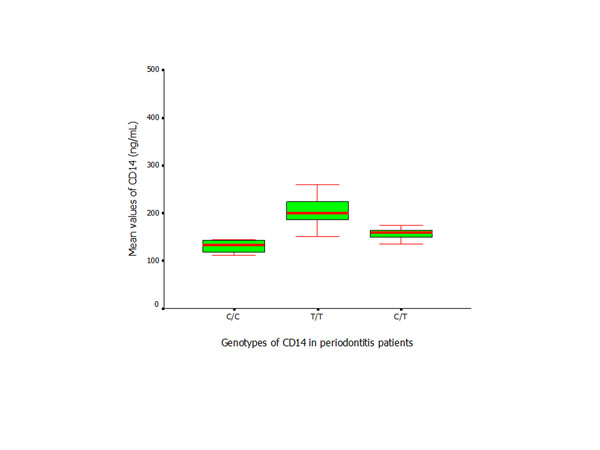
ELISA assay of CD14 (Mean ± SD) applied to measure the serum levels in periodontitis patients with different *CD14* genotypes.

The serum levels of CD14 in the patient group were significantly higher than those in the controls (*p* < 0.01) (Figure 7). Similar results were found between the subjects with the same genotypes from the control and patient groups. Within the patient group, the subjects with T/T genotype exhibited higher levels of *CD14* than subjects with C/C or C/T genotypes (*p* < 0.05), while no significant difference was found in the control subjects who presented with different *CD14* genotypes (Figures 8 and 9).

## Discussion

hBD-1 is a cysteine-rich, cationic antimicrobial peptide with broad-spectrum antimicrobial activity [19]. Thus, rationally, alterations due to genetic polymorphisms at SNP sites may be associated with asthma, HIV-1 in pregnant women and their children, as well as oral Candida carriage [51-53]. The association between *DEFB1* gene polymorphism and periodontitis could not be established in previous studies [34]. The present study, however, demonstrated that the *DEFB1* SNP at genomic position -1654, which leads to a G to A substitution and a valine to isoleucine substitution at polypeptide position [35], was present more frequently in patients with moderate to severe chronic periodontitis than the periodontally healthy group. The A/A genotype is mostly presented in the group of chronic periodontitis patients, while the G/G genotype was exhibited in the control group. It has been suggested that the genotypic make-up should be constant in an individual, and any difference in polymorphic status of the genes may arise only in circulation of the body [54].

The results of hBD-2 in serum expression support our findings from the digests. The periodontally healthy control group presented with a lower level (102.83 pg/ml) of hBD-2 than the patient group (252.09 pg/ml). The results suggest that subjects with the 1654AA genotype may tend to exhibit a higher serum level of hBD-2 in response to periodontal infection. Further investigations would be required to confirm this hypothesis.

Schaefer *et al*’s findings provide evidence for a significant association of the rare A allele of the *DEFB1* variant with an increased risk for periodontal diseases [55]. This association was independent of the periodontitis-specific covariates: smoking, diabetes and gender. The robustness of the data was further supported by a separate analysis of the two parodontopathic distinct forms: chronic and aggressive periodontitis [55]. Their results were comparable to our findings with a higher rate of A genotype and A allele and a lower rate of G genotype and G allele. The *DEFB1* indicated that the G-A nucleotide transition could influence the putative binding site of its proteins [55].

The *CD14*-159 C > T polymorphism, a SNP at position -159 in the promoter region of the gene encoding this pattern recognition receptor, is associated with elevated plasma/serum concentrations of soluble CD14, an increased risk for myocardial infarction and a decreased risk for allergies and asthma [56].

*CD14* along with TLR-4 and MD-2 detects bacterial LPS and causes the release of cytokines and antimicrobial peptides like hBDs. The present study extended to investigate *CD14* gene polymorphisms along with antimicrobial peptide polymorphisms. In previous studies, it was proven that there would be increased soluble sCD14 levels in the saliva and serum of patients with periodontal infection [57,58]. Currently, *CD14* gene polymorphisms are considered as potential diagnostic markers for periodontitis. The results from this study established the detection frequency of *CD14* T/T genotype in periodontitis patients (43%) to be significantly greater than periodontally healthy controls (26%). It is interesting to note the serum levels of CD14 in the periodontitis group was significantly higher than the levels in the controls. Similarly, periodontal patients with the same genotypes displayed higher levels of CD14 than their healthy subjects. Even within the periodontitis group, subjects with the T/T genotype exhibited higher levels of CD14 than subjects with C/C or C/T genotypes. The current findings on an increased detection frequency of T/T genotype in parallel with an increased serum level of CD14 protein is consistent with other studies on genetic polymorphisms of *CD14* gene where the T/T genotype is also shown to be more frequent in severe periodontitis. The present study supports the notion that a T/T genotype at the -159 position of the *CD14* gene is associated with chronic periodontitis. It is estimated that individuals with the T/T genotype are at twice a greater risk for moderate to severe periodontitis than people with C/C and C/T genotypes.

## Conclusions

The current study demonstrates that patients with moderate to severe chronic periodontitis present more commonly with the -1654 A/A genotype on the *DEFB1* gene and the -159 T/T genotype on the *CD14* gene, in parallel with increased serum levels of hBD-2 and soluble CD14. Within the limitations of the study, the present findings suggest that *DEFB1* and *CD14* gene polymorphisms are significantly associated with chronic periodontitis and could possibly be potential markers for assessment of risk for periodontal disease. Further investigation is warranted to elaborate on the diagnostic values of these potential markers in clinical practice.

## Competing interests

We declare that we have no financial and personal relationships with other people or organizations that can inappropriately influence our work. There is no professional or other personal interest of any nature or kind in any product, service and/or company that could be construed as influencing the position presented in, the article entitled, “Clinical application of human *β-defensin* and *CD14* gene polymorphism in evaluating the status of chronic Inflammation”.

## Authors' contributions

WTYL conducted the research, performed data collection and data analysis, and participated in manuscript writing. YY and CF, JL and YT conducted the clinical examination and performed data collection. LB performed data collection and data analysis. MW supervised clinical examination and participated in manuscript planning. HL performed data analysis and participated manuscript writing. MNBC conducted the clinical examination and participated in manuscript writing. LWCC participated in manuscript planning and writing. Dr. Liu Qing was responsible for data collection. All authors read and approved the final manuscript.
